# Public health round-up

**DOI:** 10.2471/BLT.23.011223

**Published:** 2023-12-01

**The destruction of the Gaza Strip**

A crater left by an Israeli airstrike on the Gaza Strip, where the ongoing conflict between the Israeli Defense Force and Hamas is compounding challenges faced by residents that include a health system crippled by direct assaults, a lack of medical supplies, food, water and fuel. On 12 November the World Health Organization joined partners in calling for an immediate end to attacks on hospitals in the besieged enclave.

**Figure:**
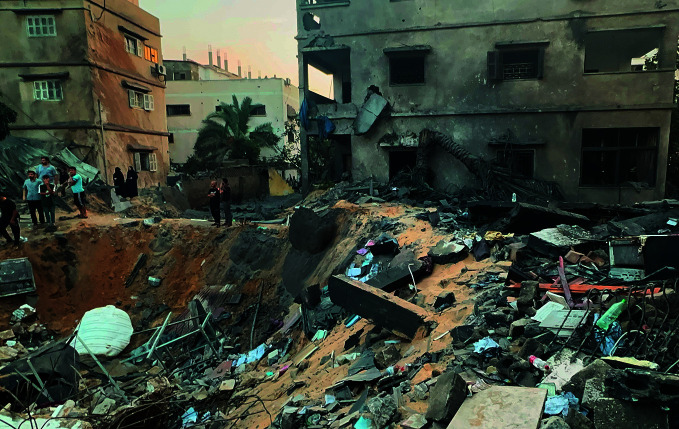
©WHO

## Conflict in the occupied Palestinian territory

The conflict in the occupied Palestinian territory (oPt) intensified with a combination of air strikes and a ground offensive in Gaza launched by the Israeli Defense Force on 27 October. According to the oPt Ministry of Health, as of 10 November at least 11 078 people had been killed since the beginning of hostilities, 41% (4506) of them children. Some 1.5 million people had been displaced. The Israeli authorities continued to restrict entry of essential supplies, as well as the entry and exit of humanitarian workers and evacuation of the injured and sick.

A further 168 Palestinians were reported to have been killed in the West Bank, where increased settler and military violence included an airstrike on the city of Jenin. Checkpoints were being blocked between oPt towns and several communities had been cut off, restricting the movement of patients, health personnel and ambulances.

According to Israeli authorities, over 1200 Israelis and foreign nationals had been killed, approximately 5400 injured and 239 held hostage since the Hamas attack carried out in Israel on 7 October.

On 12 November, the regional directors of the United Nations Population Fund, the United Nations Children’s Fund and the World Health Organization (WHO) called for urgent international action to end the ongoing attacks on hospitals in the Gaza Strip, pointing out that attacks on medical facilities and civilians are a violation of international humanitarian and human rights law and conventions.

The three agencies referenced reports of attacks on and in the vicinity of Al-Shifa hospital, Al-Rantissi Naser Paediatric hospital, Al-Quds hospital, and others in Gaza city and northern Gaza, lamenting the many fatalities including deaths of children. They also drew attention to the intense hostilities surrounding several hospitals in northern Gaza that were preventing safe access for health staff, the injured and other patients.

WHO has recorded at least 137 attacks on health care in the Gaza Strip, resulting in 521 deaths and 686 injuries, including 16 deaths and 38 injuries of health workers on duty.

https://bit.ly/3ujp8JM

https://bit.ly/46dpejq

## United Nations aid worker deaths in the Gaza Strip

The death of a United Nations Relief and Works Agency (UNWRA) aid worker in military assaults on the northern Gaza Strip on 18 November, brought to 104 the number of UNRWA personnel killed since the beginning of the conflict in the occupied Palestinian territory. This is the highest number of United Nations aid workers killed in a conflict in the history of the United Nations. 

Several incidents impacting UNRWA installations and Internally Displaced People (IDPs) had been recorded by UNRWA the day the aid worker was killed, resulting in the killing and injuring of many IDPs sheltering in the installations. As of 19 November, UNRWA was still trying to verify the correct number of casualties.

https://bit.ly/3R6Vek3

## Nepal earthquake

A 6.4 magnitude earthquake hit the Jajarkot District of Karnali Province in western Nepal on 3 November 2023. The quake was followed by a series of aftershocks, forcing people out of their homes in sub-zero temperatures. As of 6 November 2023, 153 people were reported to have died and more than 338 injured.

The ministry of health and population, WHO and other partners met to organize logistics and human resources support for the response. The government’s initial rapid assessment was launched on 5 November.

WHO has supported initial response efforts by providing interagency emergency health kits, trauma and emergency surgery kits, and medical camp kits. Dedicated hospitals were set up in Nepalgunj in the Banke district to provide treatment.

https://bit.ly/475xOlE

## Nigeria tackling human papillomavirus

Nigeria introduced the human papillomavirus (HPV) vaccine into its routine immunization system, aiming to reach 7.7 million girls, the largest number in a single round of HPV vaccination in the African region to date.

Girls aged 9–14 years will receive a single dose of the vaccine, which is highly effective in preventing infection with HPV types 16 and 18 that are known to cause at least 70% of cervical cancers.

In Nigeria, cervical cancer is the third most common cancer, and the second most frequent cause of cancer deaths among women aged between 15 and 44 years. In 2020 – the latest year for which data is available – the country recorded 12 000 new cases and 8000 deaths from cervical cancer.

https://bit.ly/40z8nGM

## Climate-resilient health framework

WHO unveiled a new operational framework for building climate-resilient and low-carbon health systems. Released ahead of the United Nations Framework Convention on Climate (COP-28), which runs from 30 November until 12 December 2023, the comprehensive Framework is designed to enhance the resilience of health systems while simultaneously reducing greenhouse gas emissions to help safeguard the health of communities worldwide.

The Framework was developed following the request for WHO support in building climate-resilient and low-carbon sustainable health systems made by health ministers from over 75 countries.

https://bit.ly/4791vSJ

## Artificial intelligence for health

WHO released a new publication listing key regulatory considerations relating to artificial intelligence (AI) for health. While AI has the potential to transform the health sector, AI technologies are being rapidly deployed, sometimes without a full understanding of how they may perform, and AI systems accessing sensitive personal information require robust legal and regulatory frameworks to safeguard privacy and security.

Released on 19 October, the publication emphasizes the importance of establishing AI systems’ safety and effectiveness, and fostering dialogue among stakeholders, including developers, regulators, manufacturers, health workers and patients.

https://bit.ly/49GfvW2

## New essential diagnostics list

WHO released its 2023 essential diagnostics list, an evidence-based register designed to support governments in procurement of diagnostic technologies.

Released on 19 October, this year’s list includes three tests for hepatitis E virus (HEV), including a rapid test to aid in the diagnosis and surveillance of HEV infection, and a recommendation regarding the use of personal glucose monitoring devices with a view to supporting better disease management.

https://bit.ly/475h0LK

## Risks of working in the sun

Nearly 1 in 3 deaths from non-melanoma skin cancer are caused by working in the sun. This is according to joint estimates published by WHO and the International Labour Organization on 8 November.

According to the estimates, 1.6 billion people of working age (15 years or older) were exposed to solar ultraviolet radiation while working outdoors in 2019, equivalent to 28% of all working-age people. In 2019 alone, almost 19 000 people in 183 countries are estimated to have died from non-melanoma skin cancer due to having worked outdoors in the sun.

https://bit.ly/3sl6qRJ

**Cover photo**

A child looks from the window of a house partially destroyed by aerial bombardment in the city of Rafah, Gaza Strip.

**Figure:**
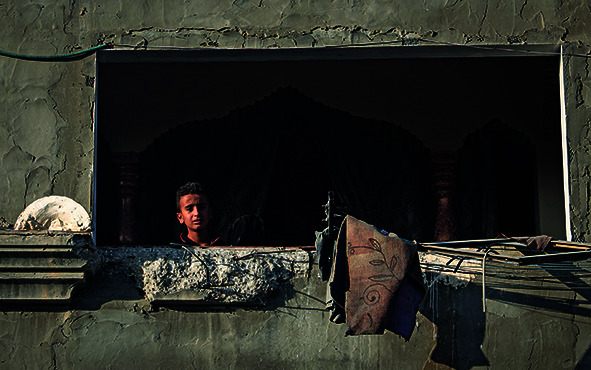
UNICEF/El Baba

## Tuberculosis control recovers

After two years of coronavirus disease 2019 (COVID-19)-related disruptions to tuberculosis (TB) control programmes, there was a significant increase in the number of people diagnosed with TB and treated in 2022.

According to the 2023 edition of WHO's global TB report which was released on 7 November, the reported global number of people newly diagnosed with TB was 7.5 million in 2022. This is the highest number since WHO began global TB monitoring in 1995, above the pre-COVID-19 baseline (and previous historical peak) of 7.1 million in 2019, and up from 5.8 million in 2020 and 6.4 million in 2021.

The report is based primarily on data gathered by WHO from national ministries of health in annual rounds of data collection.

https://bit.ly/3SB1eUm

## Developing vaccines for endemic pathogens

To facilitate and inform priorities in global vaccine development for endemic pathogens, WHO has commissioned the drafting of 16 "vaccine value profiles" (VVPs) which, once completed; are to be published in the journal *Vaccine*.

The initiative is the result of collaborations with pathogen and vaccine experts led by the product development and research team in WHO’s immunization, vaccines and biologicals department. The main object of the initiative is to support the development of vaccines for pathogens that pose a substantial public health and socio-economic burden, especially in low- and middle-income countries.

https://bit.ly/3SzMCEP

**Looking ahead**

30 November–12 December 2023. COP28 Health Pavilion: Dubai, United Arab Emirates. https://bit.ly/3PP1k7L

11–13 December 2023. WHO Meeting on Advancing the Global Influenza Surveillance and Response System. Abu Dhabi, United Arab Emirates. https://bit.ly/3ML9j55

12–13 December 2023. Tallinn Charter 15th Anniversary Health Systems Conference. Tallinn, Estonia. https://bit.ly/46cOvKN

